# The impact of benign tissue within cancerous regions in the prostate: Characterizing sparse and dense prostate cancers on whole-mount histopathology and on multiparametric MRI

**DOI:** 10.1016/j.mri.2024.110233

**Published:** 2024-09-10

**Authors:** Olga Starobinets, Jeffry P. Simko, Matthew Gibbons, John Kurhanewicz, Peter R. Carroll, Susan M. Noworolski

**Affiliations:** a Department of Radiology and Biomedical Imaging, University of California, San Francisco, San Francisco, CA 94143, USA; b The Graduate Group in Bioengineering, University of California, San Francisco and Berkeley, Berkeley, CA 94720, USA; c Department of Pathology, University of California, San Francisco, San Francisco, CA 94143, USA; d Department of Urology, University of California, San Francisco, San Francsico, CA 94143, USA; e UCSF Helen Diller Family Comprehensive Cancer Center, University of California, San Francisco, San Francisco, CA 94143, USA

**Keywords:** Prostate cancer, Multiparametric MRI, Histopathology, Prostatectomy

## Abstract

**Purpose:**

To establish the incidence, size, zonal location and Gleason Score(GS)/Gleason Grade Group(GG) of sparse versus dense prostate cancer (PCa) lesions and to identify the imaging characteristics of sparse versus dense cancers on multiparametric MRI (mpMRI).

**Methods:**

Seventy-six men with untreated PCa were scanned prior to prostatectomy with endorectal-coil 3 T MRI including T2-weighted imaging, diffusion-weighted imaging and dynamic contrast-enhanced MRI. Cancerous regions were outlined and graded on the whole-mount, processed specimens, with tissue compositions estimated. Regions with cancer comprising <50 % and ≥ 50 % of the tissue were considered sparse and dense respectively. Regions of interest (ROI) were manually drawn on T2-weighted MRI. Within each patient, area-weighted ROI averages were calculated for each imaging measure for each tissue type, GS/GG, and sparse/dense composition.

**Results:**

A large number of cancer regions were identified on histopathology (*n* = 1193: 939 (peripheral zone (PZ)) and 254 (transition zone (TZ))). Thirty-seven percent of these lesions were sparse. Sparse lesions were primarily low-grade with the majority of PZ and 100 % of TZ sparse lesions ≤GS3 + 3/GG1. Dense lesions were significantly larger than sparse lesions in both PZ and TZ, *p* < 0.0001. On imaging, 246/45 PZ and 109/8 TZ dense/sparse 2D cancerous ROIs were drawn. Sparse GS3 + 3 and sparse ≥GS3 + 4 cancers did not have significantly different MRI intensities to dense GS3 + 3 cancers, while sparse GS3 + 3/GG1 cancers differed from benign, *p* < 0.05.

**Conclusion:**

Histopathologically identified prostate cancer lesions were sparse in 37 % of cases. Sparse cancers were entirely low grade in TZ and predominantly low-grade in PZ and generally small, thus likely posing lower risk for spread and progression than dense lesions. Sparse lesions were not distinguishable from dense lesions on mpMRI, but could be distinguished from benign tissues.

## 1. Introduction

Prostate cancer (PCa) is extremely common; one in eight men in the United States expects to receive a prostate cancer diagnosis during his lifetime [1]. However, not all prostate cancers are created equal, some are aggressive and require treatment, while others grow slowly and can be safely left untreated [2,3]. It is therefore important to be able to separate clinically significant tumors requiring treatment from slow-growing indolent PCa that can be actively watched without immediate treatment.

Prostate biopsies are inherently limited by inadequate tumor sampling and frequently fail to convey the full extent of the tumors’ aggressiveness, thus providing limited confidence when it comes to treatment recommendations [4,5]. Multiparametric magnetic resonance imaging (mpMRI) is a noninvasive technique that has been gaining traction in detection and localization of prostate cancer [4–12]. By evaluating the prostate gland in its entirety, mpMRI sidesteps the greatest limitation associated with the needle biopsies. However, mpMRI comes with its own set of limitations. A major challenge in PCa characterization is posed by the heterogeneity of prostatic tissues. Aside from histologically assigned Gleason grading, which is traditionally used to estimate cancer aggressiveness, prostate cancers can also be categorized as dense or sparse based on the amount of cancerous and normal tissues within the lesion [13]. The inability to detect sparse cancers on imaging is often cited as one of the limitations associated with mpMRI of the prostate [10]. Some groups have studied the incidence and the MRI characteristics of sparse lesions, but none have provided a detailed characterization of their incidence, sizes, Gleason Grades and zonal location. Therefore, the purpose of the study was 1) to establish the incidence, size and Gleason Score (GS) / Gleason Grade Group (GG) of sparse peripheral zone (PZ) and transition zone (TZ) PCa lesions on whole-mount histopathology and 2) to identify the imaging characteristics of sparse cancers on mpMRI modalities.

## 2. Material and methods

### 2.1. Patients

This study was approved by the Committee on Human Research at this institution and was compliant with the Health Insurance Portability and Accountability Act, with participants providing written, informed consent. Seventy-six patients who underwent radical prostatectomy for a biopsy-proven prostate cancer were studied. Additional inclusion criteria were: 1) no treatment for their prostate cancer prior to surgery, 2) surgery performed less than a year after MRI, 3) cancerous regions identified on histopathology, and 4) the MRI exam was performed greater than 6 weeks after prostate biopsy to avoid biopsy artifact in the MRI. The mean age of this cohort was 63.7 ± 6.3 years and the mean PSA 6.9 ± 4.8 ng/dL. The average time between MRI and surgery was 58 ± 54 days, with a range of 2–201 days.

### 2.2. MR Imaging

All patients were imaged on a 3 T MR scanner (GE Healthcare, Chicago, IL, USA) from 2007 to 2014, with an expandable balloon endorectal coil (MedRad, Bayer HealthCare LLC, Whippany, NJ) combined with an external anterior and posterior phased array coil (GE Healthcare, Chicago, IL, USA). A perfluorocarbon fluid (Galden; Solvay Plastics, West Deptford, NJ, USA) was used to inflate the balloon coil. The patients did not receive Buscopan (Sanofi, Reading, UK) or another antispasmodic. T2-weighted images were acquired in an oblique axial plane with a fast spin echo sequence, FOV = 18 cm, slice thickness = 3 mm, matrix = 512 × 512, and TR/TE = 6000/96 ms. Diffusion weighted images (DWI) were acquired using a 2D single-shot spin echo sequence with TR/TE = 4000/78–90 ms, b = 0 and 600 s/mm^2^, slice thickness = 3 mm and either a pixel bandwidth = 1952 (conventional acquisition) or a pixel bandwidth = 1305 (reduced-field-of-view acquisition) [14]. DCE MRI was acquired using a 3D fast spoiled gradient recalled sequence with TR/TE = 3.5/0.9 ms, flip angle = 5°, slice thickness = 3 mm slices, and a single-dose (0.1 mmol/Kg) of gadopentetate dimeglumine (Gd-DTPA) (Bayer, Whippany, NJ), administered via a power injector (Medrad, Bayer, Whippany, NJ). The DCE MRI had a temporal resolution median = 10 s, range = 7–13 s, and spanned approximately 5 min. Further details have been reported previously [9]. T2-weighted images were corrected for the inhomogeneous reception profile due to the combination of the endorectal coil and the external phased array [15]. In-house software was used to generate apparent diffusion coefficient (ADC) maps from the combined DWI (b = 600 s/mm^2^) and T2-weighted reference images (b = 0 s/mm^2^). DCE MRI semi-quantitative maps were created based on the tissue enhancement parameters of peak enhancement, maximal enhancement slope, and washout rate [16].

### 2.3. Whole mount histopathology

Following prostatectomy, all prostate specimens were fixed using injected neutral-buffered formalin for at least 24 h. The prostates were then serially cross-sectioned from apex to base at 3 mm intervals using a conventional deli slicer (Hobart, Troy, OH, USA). Slices, prepared as whole-mount sections, were then embedded in paraffin, cut at 4 μm thickness, and stained with hematoxylin and eosin. The study pathologist (13 years of experience) then examined the slides under light microscopy, marking the regions of interest. Following the pathology review, the slides were digitally scanned using a standard, flatbed scanner, at ≥300 dpi for comparison to the MR images.

### 2.4. Identifying Regions of Interest

**Fig. 1.**
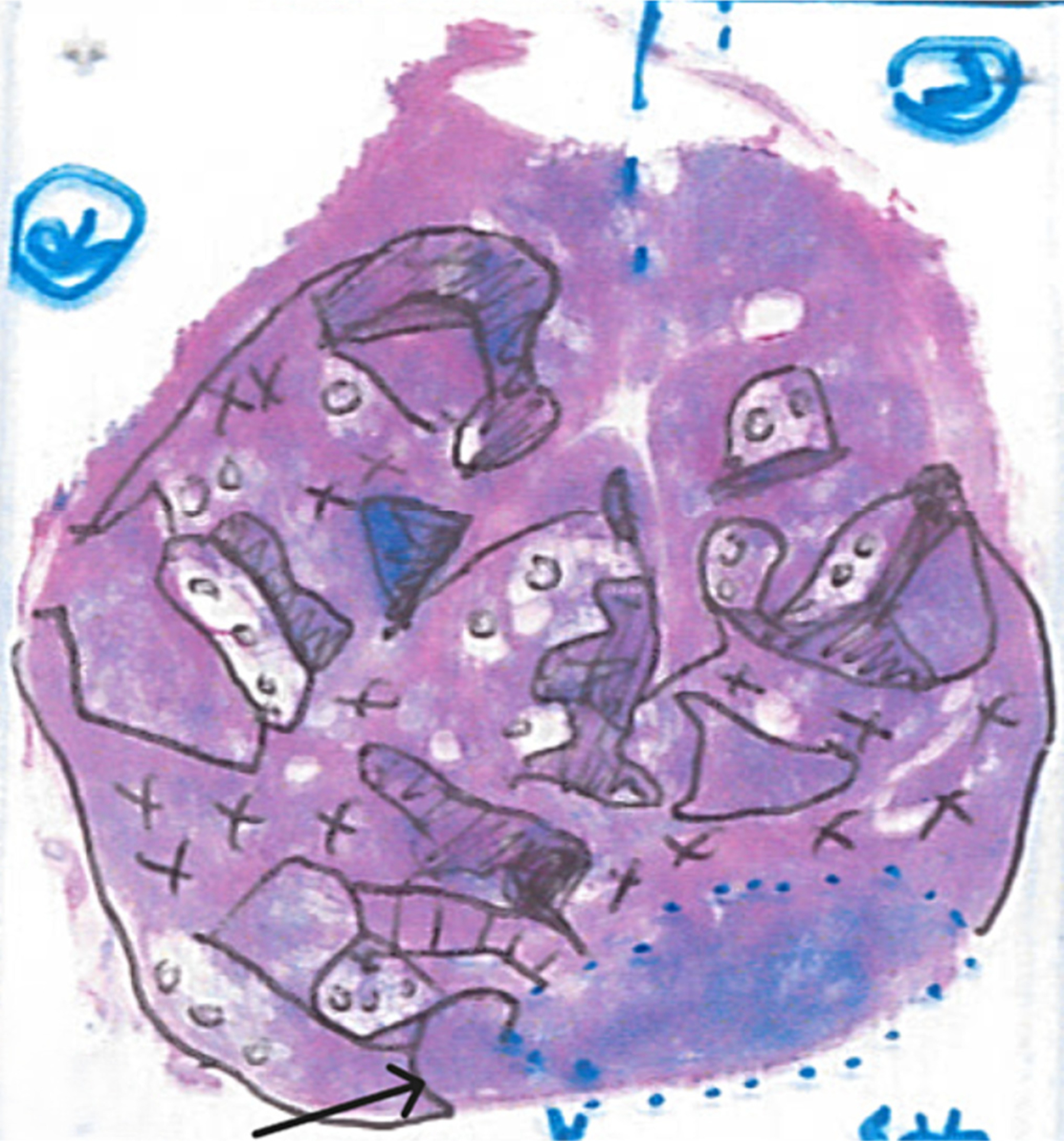
Example histopathology (hematoxylin and eosin) slide demonstrating the cancerous and benign tissues identified. Circles represent regions of cystic atrophy, slashes=stroma, ×’s=atrophy, filled in areas = inflammation; the arrow points to high grade prostatic interepithelial neoplasia and the blue dotted region is cancer with 60% cancer (G4(90%)+G3(10%)), 30% stroma and 10% glandular tissue.

The study pathologist outlined cancerous regions on each hematoxylin and eosin (H&E) stained slide and graded them using the Gleason system [17]. For each region containing cancer, the amount of cancer glands, benign glands, high-grade prostatic intraepithelial neoplasia (HGPIN) glands and stroma were estimated as percentages of regional area, along with the percentages of each Gleason grade present. In addition to cancer, regions of benign tissues (cystic atrophy, atrophy, stroma, and inflammation) were also recorded, so that all tissue types on every slide were accounted for (See Fig. 1). The normal tissues were defined to include: 1) in the PZ - normal PZ tissues and cystic atrophy located in the PZ; and 2) in the TZ - normal TZ tissues and cystic atrophy located in the TZ. The benign tissues were defined to include: 1) in the PZ – normal PZ tissues, cystic atrophy, atrophy, and inflammation located in the PZ; and 2) in the TZ – normal TZ tissues, cystic atrophy, atrophy, inflammation, and benign prostatic hyperplasia located in the TZ.

Using anatomical landmarks, ROIs were manually drawn on the T2-weighted images based on the digitized histopathology slides. The MRI images were obtained in the axial plane of the prostate to better correspond to the slice orientation of histopathology slides. Digitized, whole-mount histopathology slides were generated through the prostate. Anatomic cues, such as focal glandular BPH nodules, and borders of PZ to TZ, were used to manually determine the correspondence of MRI slices to the histopathology slides. The manually drawn ROI locations were constrained to homogeneous, artifact-free regions and were based on agreement between two readers in consensus to further reduce any errors in alignment. In addition to the pathologist-defined ROIs, ROIs were also drawn in artifact-free regions of the obturator muscle for normalization of T2-weighted MRI intensities and DCE MRI peak enhancement.

### 2.5. Histopathology Segmentation

A semiautomatic in-house software written in Matlab (MATLAB, 2016a, The MathWorks Inc., Natick, MA, USA) was used to identify the outlines of the individual cancer regions from the digitized histopathology slides. Following cancer region identification, user initialized gradient vector flow [18–20] contouring was used to segment each cancer region. Next, region areas were computed first in pixels and then converted to cm^2^. To obtain the in vivo cancer region areas, a scaling factor of 1.092 was applied to the computed area values to account for global shrinkage of resected tissue due to the histopathology fixation and processing [21].

### 2.6. Defining Sparsity and Identifying Sparse Lesions

#### 2.6.1. Histopathology

Cancerous regions outlined on histopathology slides were classified as dense if 50 % or more of the cross-sectional area was occupied by cancer; conversely, sparse cancerous regions were regions with less than 50 % of the cross-sectional area occupied by cancer, as described by Langer et al. [13]. In other words, the cancerous regions were generally mixtures of cancerous and benign tissues. “Dense” signified that cancer dominated the mixtures, whereas “sparse” cancerous regions were mixtures of benign and cancerous tissues in which the benign tissue dominated, i.e. the cancer was sparsely infiltrative. Cancerous regions were grouped into 3D lesions based on their locations on the adjacent histopathology slices. Assigning the overall sparsity and GS to the lesion was done by two different methods. 1) “Purely sparse” lesions were defined as lesions containing only sparse cancerous regions. Any lesion containing a dense cancerous region was classified as dense. 2) “Overall sparse” lesions were defined as lesions containing less than 50 % overall cancer, whether or not they had some denser areas within them. The GS of the lesion was computed as a cumulative result of all the cancerous regions included within the lesion weighted by the area of each cancerous region, since the individual Gleason Grade percentages were available for every cancerous region of interest. Based on the GS, Gleason Grade Groups (GG) (1–5) were also assigned based upon the primary and secondary Gleason Grade of the lesion, with GS ≤3 + 3 = GG 1; GS3 + 4 = GG 2; GS4 + 3 = GG 3; GS8 (4 + 4, 3 + 5 or 5 + 3) = GG 4; and GS > 4 + 4 = GG 5 [22]. Note, as Gleason Grade 2 labels were provided at the time of this study, they were included here in these very low aggressive lesions to further characterize the lesions, although Gleason pattern 2 is no longer in use [22]. For each lesion a lesion volume was computed by multiplying the total area of the included cancerous regions by the thickness of the cross-sectional prostate slices (3 mm).

#### 2.6.2. mpMRI

In the context of imaging studies, sparsity within individual ROIs was defined in the following way: ROIs with cancer occupying 50 % or more of the cross-sectional area were considered dense, while ROIs with cancer occupying less 50 % of the cross-sectional area were considered sparse. For each patient, ROIs were grouped based on the location within the prostate (PZ/TZ), tissue type (e.g. cancer, cystic atrophy, etc), GS/GG (when applicable), and dense/ sparse classification. For each of these groups, a weighted average was calculated using ROI areas for each imaging measure. For example, the mean ADC of the PZ 3 + 3 sparse lesions for a patient was calculated as the weighted average of all ADC measures in all PZ 3 + 3 sparse lesions, with each lesion’s value weighted by its corresponding ROI area. For comparisons across patients, normalizations were performed to reduce interpatient biases. T2-weighted MRI intensities were normalized by the mean signal in a manually drawn ROI in the muscle. DCE MRI peak enhancement was normalized by multiplying by 150 % divided by the enhancement in the muscle. DCE MRI maximal enhancement slope and washout slope were normalized by dividing the original signal by the mean peak enhancement in the prostate, as described elsewhere [23].

### 2.7. Statistical analysis

Statistical analysis was carried out using JMP software (JMP, Version 17, SAS Institute Inc., Cary, NC). A *p*-value of 0.05 or less was used to define statistical significance. Analyses were done separately for tissues in the peripheral zone and for tissues in the transition zone. Descriptive statistics were listed as mean ± standard deviation when normally distributed. Two-tailed, Student’s *t*-tests were used to compare the sizes of dense and sparse lesions on histopathology. Additionally, two-tailed, Student’s t-tests were used to compare the volumes of the sparse, low-grade lesions and the rest (grouping the sparse, higher-grade lesions and the dense lesions). Non-parametric Wilcoxon signed-rank tests were used to compare the imaging parameters of interest across groups of ROIs as follows. Imaging metrics for sparse tissues of differing GS/GG were compared, as well as imaging parameters for sparse tissues and dense tissues, sparse tissues and normal tissues, and finally sparse tissues and benign tissues were compared. The normal and benign tissue categories were defined above, with the normal tissue including normal and cystic atrophy while the benign tissue additionally included atrophy and inflammation.

## 3. Results

### 3.1. Histopathology

A total of 1193 cancerous regions were drawn for 76 patients: 939 cancerous regions were drawn within the peripheral zone and 254 regions were drawn within the transition zone of the prostates (Table 1). Cancerous regions ranged in size from 0.01cm^2^ to 9.27cm^2^. The zonal breakdown and the percentages of benign glandular and stromal tissues, HGPIN, and cancer are summarized in Table 1. Table 2 summarizes the GS/GG distribution found in the cancerous regions split based on the location within the prostate and the sparsity of the cancerous regions. The 1193 cancerous regions were grouped into 278 lesions; 207 lesions were classified as peripheral zone lesions, while 71 lesions belonged to the transition zone. Thirty-two patients had only PZ lesions, 3 patients had only TZ lesions and 41 patients had both PZ and TZ lesions. There was a median of 3 lesions per patient, with a range of 1–11.

First, the “purely sparse” lesions containing only sparse cancerous regions were studied. Using these criteria, there were 151 dense and 56 sparse PZ lesions (27 % sparse), 61 dense and 11 sparse TZ lesions (15 % sparse) identified. Dense lesions had similar cancer compositions across the prostate zones, with 61.36 ± 15.5 % and 63.1 ± 16.5 % cancer reported for PZ and TZ lesions. A similar cancer composition was also noted for PZ and TZ sparse lesions with 25.4 ± 9.3 % and 25.3 ± 10.9 % cancer respectively. Dense lesions were significantly larger than sparse lesions in both PZ (1.03 ± 1.77 cc > 0.065 ± 0.08 cc, *P* < 0.0001) and TZ (0.60 ± 0.8 cc > 0.07 ± 0.06 cc, *P* < 0.04). Sparse lesions were also primarily low-grade. Within the PZ, out of the 56 sparse lesions, 55 had a Gleason Score of GS3 + 3 or lower (GG 1) and only one lesion had a higher Gleason Score of GS4 + 3 + 5 (GG 3). Within the TZ, all 12 sparse lesions were GS3 + 3 / GG 1 (Table 3). The distributions of GS/GG across dense and sparse lesions in the PZ and TZ are summarized in Table 3.

Second, the “overall sparse” lesions, containing both sparse and dense cancer regions but with the overall percent cancer of less than 50 % were studied. This differs from the first approach of “purely sparse” lesions in that these sparse lesions may have foci that are dense, but also contain sufficiently large enough and sparse enough other regions to result in an overall percentage of cancer <50 % of the entire lesion. Using this “overall sparse” approach, there were 126 dense and 81 sparse PZ lesions (39 % sparse), 51 dense and 21 sparse TZ lesions (29 % sparse). Dense lesions within the PZ and TZ had similar cancer composition, with 66.4 ± 10.3 % and 68.2 ± 11.4 % reported for PZ and TZ lesions. Once again, dense lesions were statistically larger than sparse lesions in both PZ (1.12 ± 1.89 cc > 0.22 ± 0.50 cc, *P* < 0.0001) and TZ (0.64 ± 0.87 cc > 0.21 ± 0.29 cc, *P <* 0.035). Of note, the additional 35 lesions that were overall sparse as opposed to purely sparse were larger than the purely sparse lesions (median(Q1,Q3) = 0.29 (0.12,0.57) cc *>* 0.035(0.02,0.09) cc, *P* < 0.0001 (Wilcoxon)) and, in the PZ only, included higher GG lesions. The distributions of GS/GG across dense and sparse lesions in the PZ and TZ are summarized in Table 3.

**Fig. 2.**
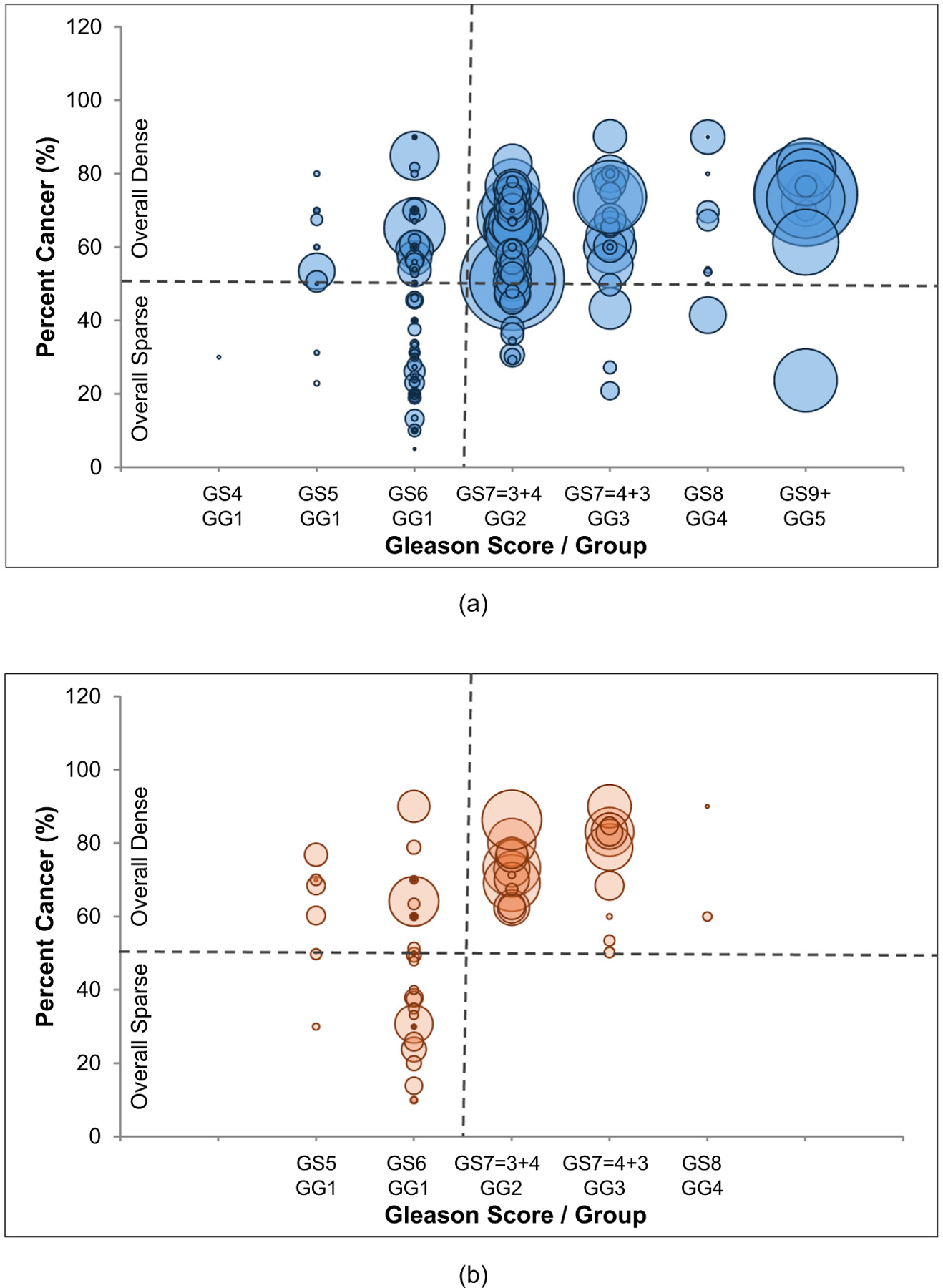
Bubble plots of 3D lesions: A. in the PZ and B. in the TZ. Overall percent cancer of the lesion is plotted against the Gleason Score / Grade Group of the lesion, with the size of the bubbles representing lesion volumes. The horizontal dotted line represents sparsity (everything below 50% cancer considered sparse (with the “overall” sparse definition)); the vertical dotted line represents aggressiveness with everything Gleason Score 6 or lower considered low-grade.

Fig. 2 depicts the relationship between the GS/GG, the overall percent of cancer, and the volumes for lesions in the PZ and the TZ. The two plots are drawn to the same scale; meaning the differences in bubble sizes represent the true differences between the lesion volumes within the peripheral and the transition zones. Lesions in the sparse and low-grade regions (lower left quadrant) for both PZ and TZ plots were smaller in volume than their dense or more aggressive counterparts (*P* < 0.0001 for PZ and *P* = 0.008 for TZ). The majority of large lesions both within the PZ and TZ were predominantly found in the dense, higher-grade quadrants (upper right quadrant). Within the TZ, there were no sparse lesions observed ≥GS4 + 3/GG3. There was one of each GS8 / GG4 and GS9 /GG5 lesions found within the sparse PZ lesions, which were 1.42 cc and 4.10 cc in volume, respectively.

### 3.2. Multiparametric MRI

On imaging, 246 dense and 45 sparse cancerous 2D ROIs were drawn in the PZ and 109 dense and 8 sparse 2D ROIs were drawn in the TZ. The smallest ROIs drawn were 0.06 cm^2^. On average, dense and sparse ROIs in the PZ were 0.82 ± 0.91 cm^2^ and 0.45 ± 0.24 cm^2^ in size with 70.9 ± 11.6 % and 29.6 ± 8.8 % of these being cancer. Within the TZ, on average dense and sparse ROIs were 0.59 ± 0.35 cm^2^ and 0.41 ± 0.21 cm^2^ in size containing 76.1 ± 9.9 % and 28.4 ± 11.6 % cancer. Table 4 lists the distribution of GS/GG across the regions of interest drawn on MR imaging in the PZ and the TZ.

These cancerous 2D regions were grouped into sparse and dense lesions. The distribution of GS/GG across sparse and dense lesions outlined on MR imaging in the PZ and the TZ is summarized in Table 5. In addition to grouping cancer regions, benign regions were grouped to yield 68 benign volumes in the PZ and 61 benign volumes in the TZ. Normal tissues regions were grouped into 62 normal tissue volumes within the PZ and 42 normal tissue volumes in the TZ.

**Fig. 3.**
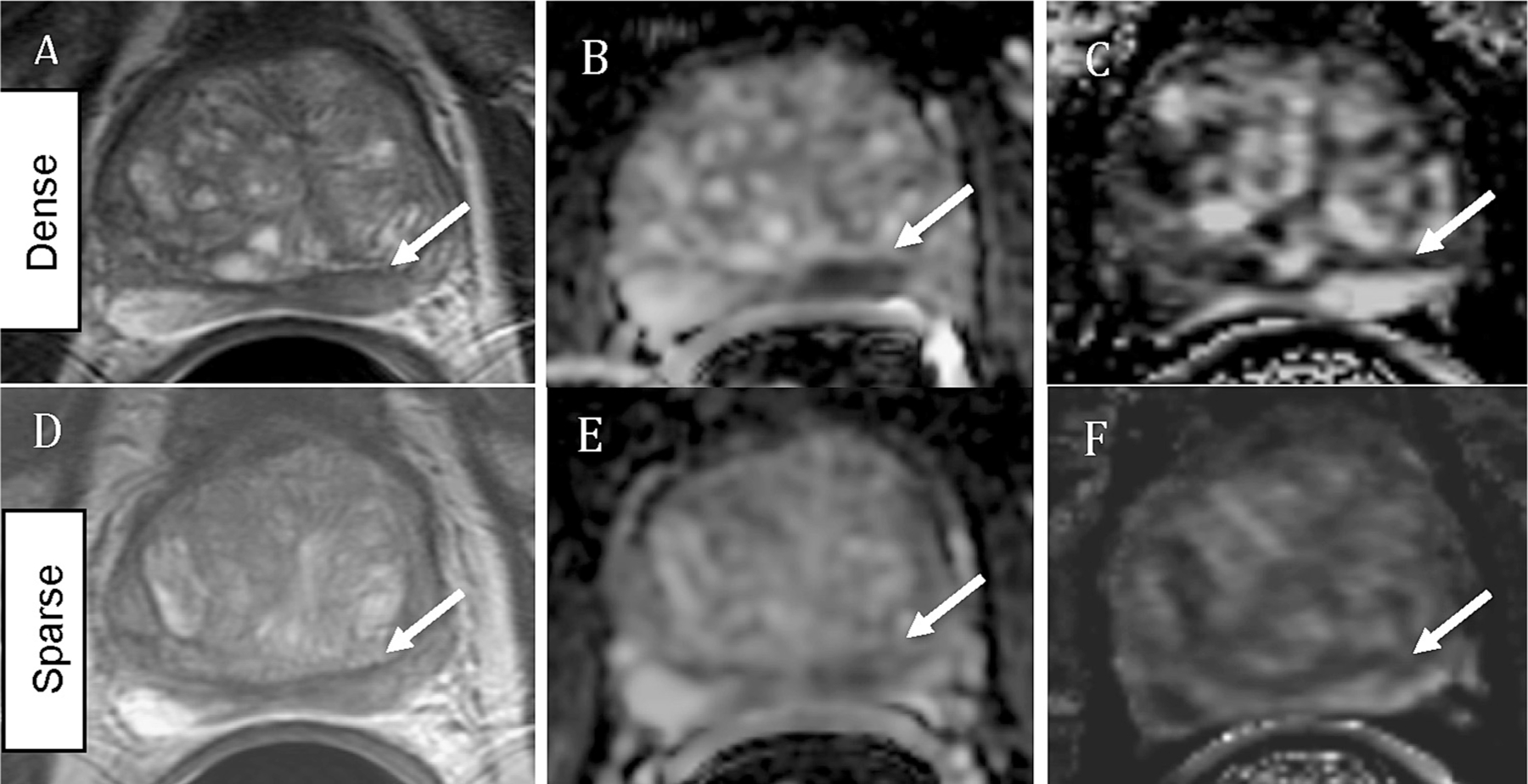
A 74 year-old male with serum PSA of 10.3ng/ml who underwent radical prostatectomy. Top panel: dense GS4+3 /GG3 cancer (60% cancer): A) coil-corrected T2-weighted image (normalized T2=3.47), B) ADC map (942 ×10–3mm2/s), C) maximal enhancement slope (157 %baseline/min). The histopathology of this slide is shown in Fig. 1. Bottom panel: sparse GS4+3 /GG 3 cancer (30% cancer): D) coil-corrected T2-weighted image (normalized T2=3.81), E) ADC map (1271 ×10–3mm2/s), F) maximal enhancement slope (132 %baseline/min). The arrows designate cancerous regions.

Fig. 3 demonstrates an example of a dense GS4 + 3 / GG3 cancer region (top panel) and a sparse GS4 + 3 /GG 3 cancer region (bottom panel) as these regions appear on mpMRI modalities: T2-weighted imaging, ADC maps, and maximal enhancement slope maps. These regions belong to the same lesion; however, while the dense region contains 60 % cancer, the sparse region contains only 30 % cancer. The cancer in the dense region appears more aggressive on all modalities than the cancer in the sparse region.

**Fig. 4.**
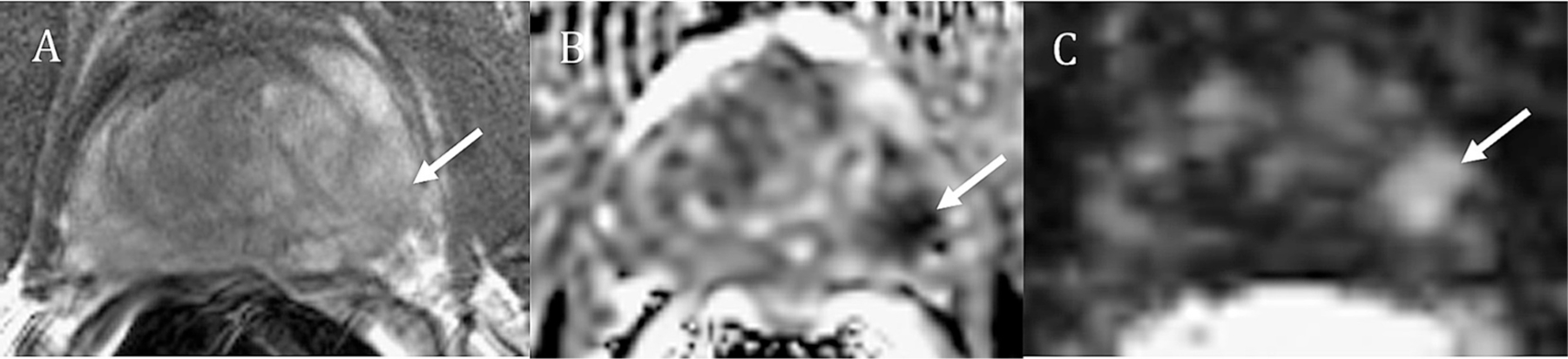
MRI of a 60 year-old male with serum PSA of 9.3 ng/ml who underwent radical prostatectomy. Sparse GS4+5 /GG 5 cancer (20% cancer) as seen on: A) coil-corrected T2-weighted image (normalized T2=1.45), B) ADC map (1114×10–3mm2/s), and C) maximal enhancement slope (149 %baseline/min). The arrows designate the cancerous region.

While the majority of sparse lesions are small and low-grade, there were exceptions. For instance, in Fig. 2A, there are two outliers in the sparse PZ region, representing two high-grade, large lesions. Fig. 4 depicts a prostate slice containing a sparse GS4 + 5 / GG5 cancerous region (20 % cancer) from the 4.10 cc GS9/GG5 outlier lesion. While the cancer may not be obvious on T2-weighted imaging, a signal reduction on the ADC map and an elevated enhancement on the maximal enhancement slope map clearly indicate the presence of malignancy. Similarly, the other outlier, a 1.42 cc sparse GS8 /GG4 lesion, was also visible on imaging.

**Fig. 5.**
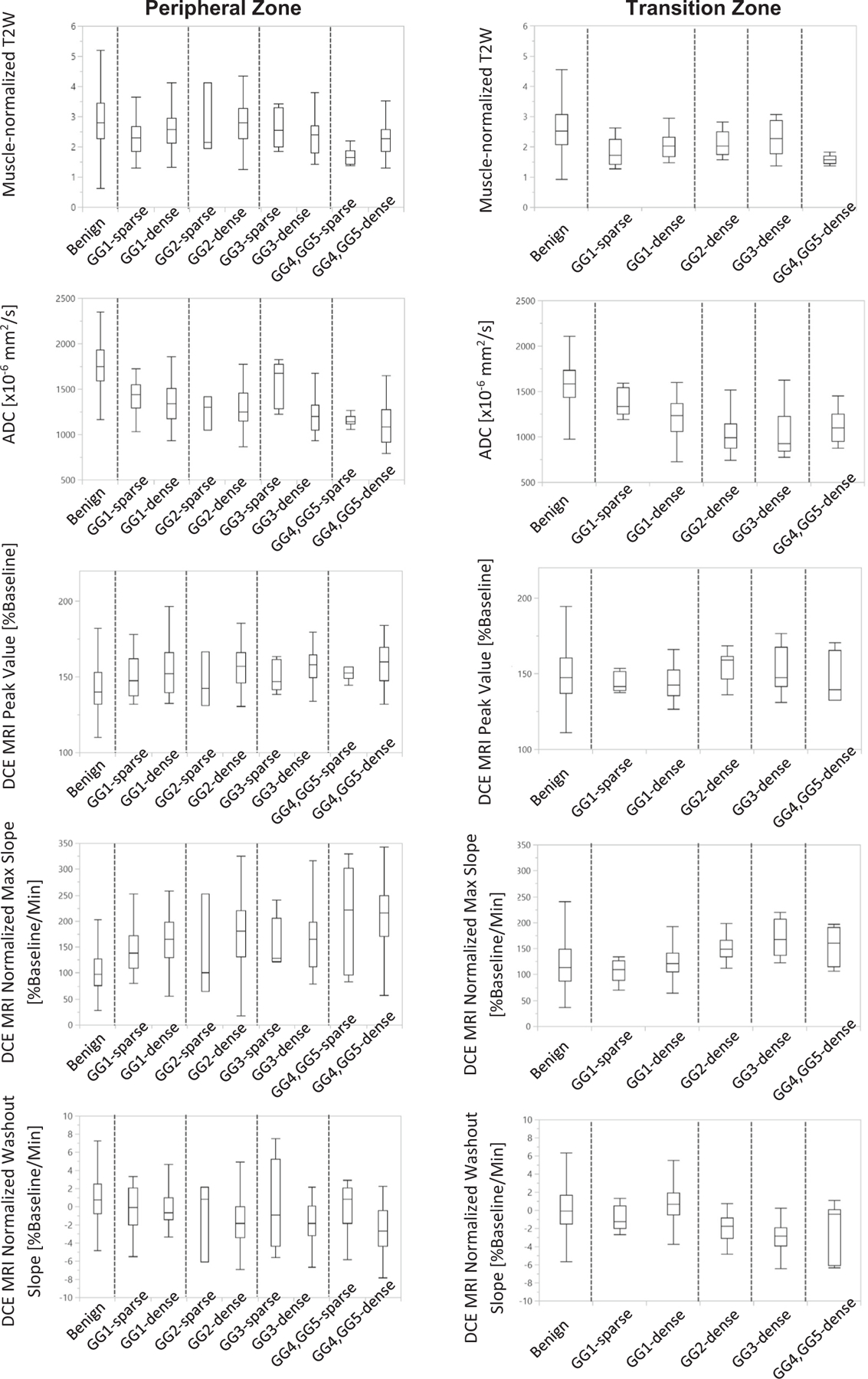
Box plots of MRI-measured intensities for benign tissues, and sparse and dense cancer individual ROIs by Gleason Score/Gleason Grade Groups in (A) the peripheral zone and (B) the transition zone.

MpMRI results were reviewed to determine whether imaging could be used to distinguish sparse cancers from other tissue types. Within the PZ, when comparing the individual ROIs, some significant differences were observed (Fig. 5). The sparse GG1 cancers were significantly lower than benign tissues on muscle-normalized T2-weighted imaging (2.31 ± 0.55 < 2.96 ± 1.02, *P* < 0.001) and ADC (1.413 ± 0.190 < 1.757 ± 0.240 × 10^−3^ mm^2^/s, *P* < 0.001) and significantly higher on DCE MRI peak enhancement (150 ± 13 > 143 ± 15 %baseline, *P* < 0.02) and DCE MRI maximal enhancement slope (142 ± 41 > 104 ± 38 %baseline/min, *P* < 0.001). The sparse GG1 cancers compared to the dense GG1 cancers were lower on muscle-normalized T2 weighted imaging (2.31 ± 0.55 < 2.64 ± 0.62, *P* < 0.03) and lower on maximal enhancement slope (142 ± 41 < 160 ± 45 %baseline/min, *P* < 0.05).

**Fig. 6.**
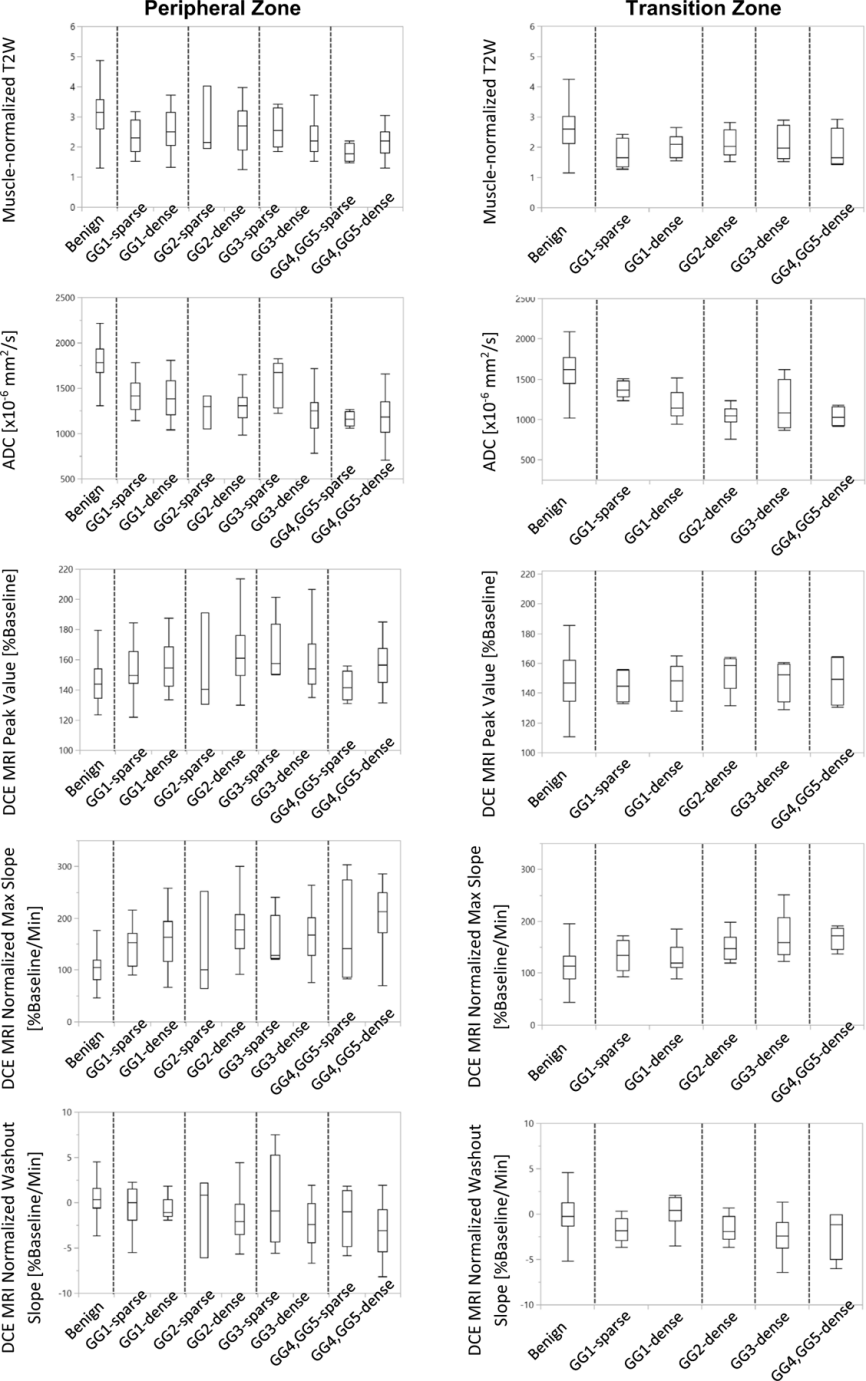
Box plots of MRI-measured intensities for benign tissues, and sparse and dense cancer lesions, after consolidation into one representative measure per tissue type per patient. Measures are plotted by Gleason Score/Gleason Grade Groups in (A) the peripheral zone and (B) the transition zone.

When the individual ROIs were combined into their respective lesions and consolidated into one representative measure per tissue type per patient, similar trends were observed, but with less significance. No statistically significant differences were found when sparse GG 1 lesions were compared to sparse GG2 lesions or to all sparse ≥GG2 lesions for any of the imaging metrics (See Fig. 6). No statistically significant differences were found between sparse and dense GG1 lesions or between sparse GG2 cancers and dense GG1 cancers for any of the parameters. Muscle-normalized T2-weighted imaging of sparse ≤GS3 + 3/GG 1 cancers (2.33 ± 0.52) was significantly lower (*P* < 0.0001) than either normal PZ tissues (2.83 ± 0.97) or benign PZ tissues (3.30 ± 1.02). Similarly, ADC of sparse ≤GS3 + 3/GG 1 cancers (1.422 ± 0.179 × 10^−3^ mm^2^/s) was significantly lower (*P* < 0.0001) than either normal PZ tissues (1.831 ± 0.197 × 10^−3^ mm^2^/s) or benign tissues (1.805 ± 0.203 × 10^−3^ mm^2^/s). DCE MRI peak enhancement was significantly higher in sparse ≤GS3 + 3/GG 1 cancers (154 ± 16 %baseline) than normal PZ tissues (145 ± 15 %baseline) or than benign PZ tissues (146 ± 15 % baseline), *P* = 0.037. DCE MRI normalized maximal enhancement slope was significantly higher (145 ± 35 %baseline/min) than in normal PZ tissues (103 ± 32 %baseline/min or benign PZ tissues (106 ± 32 % baseline/min) *P <* 0.0002.

Within the TZ, there were few sparse regions and lesions. When comparing the individual ROIs (Fig. 5), muscle-normalized T2 weighted imaging (1.82 ± 0.5 < 2.54 ± 0.85) and ADC (1.383 ± 0.157 < 1.587 ± 0.232 × 10^−3^ mm^2^/s) were significantly lower in the sparse GG1 cancers versus benign tissues (*P* < 0.03*)* and no significant differences were found between the sparse and dense GG1 cancers or between the dense GG1 and sparse GG2 cancers. When the ROIs were combined into their respective lesions and consolidated into one representative measure per tissue type per patient, the number of sparse lesions was low (*N* = 5) and included only GG1 cancers. No statistically significant differences were found between dense and sparse GG1 lesions in the TZ, although ADC had a trend to be higher in sparse cancers, (1.375 ± 0.105 > 1.197 ± 0.172 × 10^−3^ mm^2^/s, *P* = 0.058) (See Fig. 6). When comparing the ADC of sparse GG1 cancers (1.375 ± 0.105 × 10^−3^ mm^2^/s) to either normal TZ tissues (normal and cystic atrophy) (1.640 ± 0.253 × 10^−3^ mm^2^/s) or to benign TZ tissues (1.589 ± 0.225 × 10^−3^ mm^2^/s), ADC was significantly lower (*P* < 0.02). Additionally, DCE MRI normalized washout was significantly lower (*P* < 0.05) in sparse GG1 cancers (−1.72 ± 1.44 % baseline/min) than normal TZ tissues (0.20 ± 2.18 %baseline/min) or than benign TZ tissues (0.14 ± 2.38 %baseline/min).

## 4. Discussion

Prostate cancer has been the subject of intense study for decades [24]. Much is now known about its histological appearance [17,22,25–27] and imaging characteristics [6,7,9,11,23]. However, the concept of cancer sparsity when applied to prostatic lesions has not been thoroughly explored as to the relationships among the lesions’ sparsity, sizes, GGs, and zonal locations. Several groups have studied prostate cancer lesions that are mixtures of cancerous epithelial cells and other components of the tissue, with varying definitions of sparse, infiltrative or intermediate density of the cancerous lesions [13,28–32]. Many groups have investigated the question of characterizing MR undetected cancers, finding, among other aspects, that sparsity of the cancer was statistically higher in undetected lesions versus detected lesions [28–32].

Consistent with the prior study by Langer et al. [13], we defined dense regions as those with 50 % or more of the cross-sectional area occupied by cancer, while sparse cancerous regions were defined as regions with less than 50 % of the cross-sectional area occupied by cancer. Out of the 1193 cancerous regions outlined on histopathology slides obtained from 76 patients, 39 % of cancer regions in the PZ and 31 % of cancer regions in the TZ were classified as sparse. Sparse regions contained on average 25 % cancer (vs. 70 % cancer seen for dense regions) and were predominantly low-grade; 83 % of sparse PZ cancerous regions were GG1 and 95 % of sparse TZ cancerous regions were GG1. Van Houdt et al. also looked at this granular level of histology regions, finding 19 % of their 119 regions were of intermediate cancer density (which likely differs from our 50 % cutoff) as opposed to dense [32]. Their lower rate of mixed cancer/benign lesions (i.e. “sparse”) is likely primarily due to their solely analyzing MR-detected lesions, comprising 51 % of their lesions, for this analysis.

While it is informative to look at the individual, single histology-slide cancerous regions, it is also important to look at sparsity in the context of cancerous 3D lesions. Note, cancerous lesions are frequently comprised of multiple histopathological cancerous regions, as the lesions can span multiple histopathology slides. Differences in lesion composition are associated with differences in disease progression and malignant potential, as well as our ability to detect these lesions either through biopsies or imaging. Classifying lesions as sparse is not trivial and can be done in a number of ways. One approach is to characterize purely sparse 3D lesions. Another approach is to take a closer look at how sparse regions contribute to the overall composition of a 3D lesion.

Our first approach defining “purely sparse” lesions as those containing only sparse cancerous regions resulted in homogeneously sparse lesions. When viewed in this way, our prostate cancer histopathological findings suggest that on average “purely sparse” lesions are much smaller than dense lesions (~16-fold in the PZ and ~ 8-fold in the TZ) and are primarily low-grade. While the majority of such lesions were smaller than 0.1 cc in volume, the largest purely sparse lesions found in the PZ and TZ were 0.44 cc and 0.22 cc in volume, which suggests that sparse cancers as they grow do not necessarily acquire a dense core but may retain their diffuse nature. In the TZ, all 12 sparse lesions were GG1; while in the PZ, 55/56 lesions were GG1 and only a single higher-grade (GS4 + 3 + 5) lesion was identified. As small size and low-grade cancers have minimal metastatic potential [31], these histopathological findings that the purely sparse lesions are small and primarily low grade, suggest they have limited clinical significance. The patient with the single high-grade sparse GS4 + 3 + 5 lesion (0.19 cc) also had two larger, dense GG3 (1.14 cc) and GG2 (1.23 cc) lesions. In the future, it might be informative to study the progression of sparse cancers in the setting of exclusively benign tissues versus in the presence of high-grade prostatic disease.

Our second “overall sparse” approach consisted of defining sparse lesions as containing less than 50 % of cancer overall. Sparse and dense regions comprising each lesion were combined with their Gleason Score contributions weight-averaged based on the area of each region. Thus, these “overall sparse” lesions, in addition to having sparse regions, may have had a dense focus to them. Viewed in this way, 80 % of sparse lesions in the PZ and 100 % of sparse lesions in the TZ were GG1, compared to 43 % of dense lesions in the PZ and 52 % of dense lesions in the TZ being low grade. With this approach, the “overall sparse” lesions were still smaller in size than their dense counterparts but not as drastically as seen above. A Gleason 4 component identified in 16/81 (20 %) sparse lesions in the PZ points to the potential clinical significance of these lesions. The heterogeneity and the diffuse nature of these lesions reemphasizes the drawbacks of TRUS-guided biopsies that may capture a small segment of a sparse region, missing a dense or a more aggressive portion of the lesion. While the purely sparse lesions (*n* = 67) were very small (median = 0.04 cc and all <0.5 cc) and virtually all (99 %) GG1, the additional 35 lesions that were overall sparse were substantially larger (median = 0.29 cc) and, in the PZ only, included higher GG lesions. This suggests a more malignant potential for these heterogeneously sparse lesions as compared to the purely sparse lesions.

We found 24 % of our 279 lesions to be homogeneously sparse, and 37 % of lesions to be overall sparse. While others did not differentiate between the homogenously sparse and overall sparse lesions, our incidence of sparse lesions is in general agreement with others using a < 50 % cancer cutoff criteria in smaller (*n* < 45 lesions) studies (23 %–36 %) [13,29] while those who used a more encompassing definitions for infiltrative cancers predictably found higher rates, up to 62 % [28,30,31]. We found a trend for a higher rate in the PZ than in the TZ (39 % vs. 29 %). Others did not report this breakdown, except Langer et al. who only studied PZ cancers and found 36 % were sparse [13]. Our studying 279 lesions provides higher confidence in our rate as compared to the Langer et al. study of 28 lesions [13].

Several groups have investigated the question of characterizing MR undetected cancers and found, among other characteristics, that the undetected lesions were more apt to be mixtures of cancer and other components, whether luminal space or benign glandular or stromal tissue [28,29,31,32]. They also found these undetected prostate lesions were smaller and/or had lower GG, as we found in our sparse lesions [28,29,31,32].

In our study, compared to cancer regions drawn on histopathology, cancer regions drawn on MRI images were fewer in number (especially true for small, low-grade regions) and were similar in size for dense lesions but on average slightly smaller in size for sparse lesions (0.55 ± 0.71 cm^2^ on histopathology vs. 0.44 ± 0.71 cm^2^ on MR imaging). This observation underlines the fact that sparse cancers (partly due to their diffuse nature and partly due to their predominantly low grade) are not always easy to identify on imaging, consistent with others’ findings in MR-undetected prostate lesions [28,29,31,32].

Looking at imaging metrics, no statistically significant differences were found Between sparse GG1 and ≥ GG2 cancers or sparse and dense GG1 cancer lesions in the PZ. Van Houdt et al. found higher T2 and higher ADC in their intermediate density cancers versus the high density cancers but did not stratify these groups by GG or tumor morphology for which they found significant MR differences which may have driven the density results [32]. Other groups found significant MRI differences between MR-detected and MR-undetected cancers, with the undetected cancers generally having larger luminal spaces, with concomitantly higher ADC and higher T2-weighted signal intensity, consistent with a mixture of benign glandular and cancerous glandular tissue [29,32]. [29,32–35]. While their MR-undetected cancers were more apt to be sparse/infiltrative, they did not analyze their MRI data from the approach of comparing the sparse cancers versus the dense cancers, so it is unclear if they would have been able to separate these on MRI. Additionally, they did not stratify their lesions by GG and prostate zone, which may have dominated the relationships. [29,32–35] Another point to note is that cancer contribution is a continuum – there is a spread of cancer percentage in both those deemed sparse and those deemed dense, making separation of the groups challenging.

Interestingly, despite our inability to differentiate low-grade sparse and dense lesions, we did find statistically significant differences between GG1 sparse cancers and benign tissues on T2-weighted imaging, ADC, DCE MRI peak enhancement, and DCE MRI maximal enhancement slope. An explanation for this may lie in the composition of sparse regions. We showed that sparse regions were composed predominantly of stromal tissues with smaller contributions from cancer and glandular tissues. This result is consistent with Miyai et al.’s finding of a higher percent of stroma (45 %) in their MR-undetected cancers, which were generally sparse (average of 43 % cancer), versus their detected cancers [35]. Stromal tissues do not have ductal/luminal spaces and typically have a “cancer-like” MR appearance, which could explain why it is hard to distinguish between the dense and the sparse cancers on imaging [16,23,33,34]. It also explains why it is still possible to differentiate between the sparse GG1 cancers and benign tissues, which are more glandular in nature and provide a better contrast to stromal-dominated sparse cancer lesions. Of note, when comparing the individuals ROIs, the muscle-normalized T2-weighted intensity was significantly *lower* in sparse versus dense GG1 cancer, supporting this theory that the stromal tissues, with their lack of ductal/luminal water cause more of the effect of the lower T2-weighted intensity versus the effect of gland shrinkage in low grade (GG1) cancer. Others have investigated the histological characteristics driving MR measures, finding ADC and T2 are higher with greater luminal space, supporting our theories about the impact of stroma on MR differentiation of our sparse cancers from benign glandular tissues [29,32–34,36,37].

Furthermore, to address the concern of low numbers of sparse cancers in determining MRI significant differences, the individual ROIs were additionally analyzed using a cutoff of <67 % cancer for sparsity, yielding the minimum number of cases in a PZ group to be 14, while the TZ sparse cancers were still rare (14 GG1, 2 GG2 and 4 GG3). These analyses resulted in similar relationships as the <50 % cutoff data (Data not shown).

Our ability to distinguish between sparse low-grade lesions and benign tissues on mpMR imaging is of clinical significance since accurate evaluation of the extent of the disease is important for treatment planning. Sparse regions are often found at the periphery of dense cancerous regions, which are more easily detected on mpMRI [28–32]. Since effective focal treatments are impossible to achieve without identifying accurate lesion margins, our findings suggest that even very straight forward imaging metrics – T2-weighted image intensity, ADC, and semi-quantitative evaluation of DCE MRI studies (especially in the PZ) – are capable of capturing sparse PCa, albeit with the potential to also treat regions of benign stromal tissues.

Our findings in the TZ were similar. No statistically significant differences were found on imaging parameters for TZ tissues when sparse GG1 cancers were compared to dense GG1 cancers. When compared to benign or normal TZ tissues, both ADC and DCE MRI normalized washout were significantly lower in the sparse GG 1 TZ cancers. The smaller differences between the sparse cancers and benign tissues of the TZ may reflect the normally present stromal tissue in the TZ, making differentiation of cancer mixed with benign stroma from the benign tissues more difficult than in the PZ, although they were still significant. Additionally, these comparisons are limited due to the low number of sparse TZ lesions identified on MR imaging (*N* = 5).

While the majority of sparse lesions are small, low-grade, and may never progress enough to require treatment, there are exceptions. In this study, there were two large, high-grade, sparse PZ lesions – a 1.42 cc GG4 lesion and a 4.10 cc GG5 lesion. These lesions are concerning. The good news is that despite their sparsity, these high-grade lesions were detectable on mpMRI, even within a region comprising only 20 % cancer. This suggests that, despite their sparse nature, large, high-grade lesions are detectable on mpMRI imaging with the multiple parameters increasing confidence in the detection, as the sparse cancer in Fig. 3 was less conspicuous on the T2-weighted image versus on the ADC and maximal enhancement slope images. In a different case, the GG3 cancer shown in Fig. 2 appeared more aggressive on MRI in its dense (60 % cancer) region versus its sparse (30 % cancer) region. Without the knowledge of the histopathological grading, based on its appearance on imaging alone, the sparse cancer region could be confused for a low-grade disease, however, it too, was still detectable as cancer on MRI.

This study had several limitations. First, the regions of interest were manually drawn on MR images based on the histopathology; this approach likely introduced a bias toward outlining regions visible on MR, likely corresponding to more aggressive disease. Second, there was only one pathologist assessing the slides, thus histopathology results may be affected by inter-observer variability. Third, as this study was performed prior to the advent of the Prostate Imaging-Reporting and Data System (PI-RADS), ADC maps were generated from the diffusion weighted sequence using a b-value of 600 s/m^2^, slightly lower than the currently recommended 800–1000 s/m^2^ [12], which is likely to have resulted in a small increase (<10 %) in ADC for both normal and cancerous tissues, but thus little effect on cancer discrimination [38]. Fourth, MR sequences were manually aligned to each other based on visual assessment; small shifts in alignment could have taken place and influenced the results. Fifth, analyses in the TZ were confounded by the low numbers of sparse ROIs drawn on MR images. Sixth, the small number of high-grade, sparse lesions limits the ability to determine the MR-detectability of such lesions in general, although our findings suggest that this would be a rare occurrence. Lastly, while most cases had surgery less than 2 months after the MRI, four cases did have surgery more than 6 months, although less than 7 months after the MRI. While their cancers may have progressed between the MRI and surgery, this is likely to be to a minor degree, as three of these cases had GG1 cancer and one had GG2 cancer.

In conclusion, we characterized the prevalence, size, zonal location and GG of sparse cancers on histopathology, finding sparse cancers were common (36 % of 279 lesions were overall sparse), small, and were predominantly low grade (85 %). Lesions that were overall sparse, but with a dense component, were larger and included higher GG cancer than purely sparse lesions. We determined that sparse GG1 and ≥ GG2 cancer lesions did not have different MR imaging characteristics as dense GG1 cancers, but we were able to find statistically significant differences between sparse low-grade cancers and benign tissues on several MR imaging modalities in the PZ and TZ. This suggests that mpMRI may prove valuable for focal treatment planning to ensure cancer coverage even in the setting of cancer sparsity. Further study of sparse cancers on mpMRI, especially in the TZ, is needed to fully understand the limitations of imaging when it comes to sparse lesion detection. The encouraging news is that based on our histopathology findings, sparse cancers were entirely low grade (GG1) in the TZ and predominantly low-grade (80 % GG1, 93 % ≤ GG2) in the PZ and thus overall may pose limited malignant potential for spread and progression.

## Figures and Tables

**Table 1:** Number of cancer regions outlined on histopathology, the areas of cancer regions outlined on histopathology and the distribution of tissue types within dense and sparse cancer regions.

Zonal Anatomy and Sparsity	Histopathology Outlined Cancer Regions (N)	Histopathology Outlined Cancer Regions Area (cm^2^)	Tissue Types			
			Glandular (% Lesion)	Stromal (% Lesion)	Cancer (% Lesion)	HGPIN (% Lesion)
Dense PZ	571	0.84 ± 1.22	3	27	69	1
Dense TZ	176	0.69 ± 0.76	1	27	71	0
**Dense Overall**	**747**	**0.80 ± 1.14**	**3**	**27**	**70**	**1**
Sparse PZ	368	0.27 ± 0.46	11	58	25	5
Sparse TZ	78	0.25 ± 0.45	6	65	26	1
**Sparse Overall**	**446**	**0.26 ± 0.46**	**10**	**59**	**25**	**4**

Area values are displayed as mean ± standard deviation.

**Table 2:** Histopathology: dense and sparse cancerous regions separated by Gleason Score and Gleason Group.

Gleason Score	GleasonGroup	Peripheral Zone		Transition Zone	
		Dense	Sparse	Dense	Sparse
≤3 + 3^a^	1	272 (48)	305 (83)	101 (57)	74 (95)
3 + 4^b^	2	100 (18)	12 (3)	25 (14)	0 (0)
4 + 3^c^	3	79 (14)	26 (7)	32 (18)	3 (4)
3 + 5,4 + 4,5 + 3^d^	4	61 (11)	13 (4)	14 (8)	1 (1)
≥4 + 5^e^	5	59 (10)	12 (3)	4 (2)	0 (0)
**Total (N)**		**571**	**368**	**176**	**78**

Sparse cancerous regions were defined as 2D regions with cancer occupying <50 % of cross-sectional area of the region. Percentages are given in ()’s.

a Includes: 2 GS2 + 2: 1 sparse PZ, 1 dense TZ; 13 GS2 + 3: 4 dense PZ, 4 sparse PZ, 5 dense TZ; 22 GS3 + 2: 14 dense PZ, 2 sparse PZ, 6 dense TZ.

b One dense PZ and 2 dense TZ are GS3 + 4 + 5

c Six dense and 3 sparse PZ are GS 4+3+5

d Two dense PZ, 1 sparse PZ and 1 dense TZ are 3+5; 7 dense and 1 sparse PZ are 5+3

e 20 dense and 5 sparse PZ are 5+4; 15 dense PZ and 3 dense TZ are 5+5

**Table 3:** Histopathology: number of dense and sparse 3D lesions separated by Gleason Score / Gleason Group and zone.

Gleason Score		“Purely” Sparse Lesions				“Overall” Sparse Lesions			
	Gleason Group	Peripheral Zone		Transition Zone		Peripheral Zone		Transition Zone	
		Dense	Sparse	Dense	Sparse	Dense	Sparse	Dense	Sparse
≤3 + 3	1	65	55	36	11	55	65	26	21
3 + 4^a^	2	45	0	13^a^	0	35	10	13^a^	0
4 + 3^b^	3	21^b^	1^b^	10^b^	0	18^b^	4	10^b^	0
4 + 4,5 + 3^c^	4	9^c^	0	2	0	8^c^	1	2	0
≥4 + 5	5	11	0	0	0	10	1	0	0
**Total (N)**		**151**	**56**	**61**	**11**	**126**	**81**	**51**	**21**
**Patients (N)**		**71**	**30**	**42**	**7**	**65**	**40**	**37**	**13**

“Purely sparse” lesions defined as 3D lesions containing only sparse cancerous regions.

“Overall sparse” lesions defined as 3D lesions containing <50 % cancer overall.

a One dense (both categories) TZ lesion is GS 3 + 4 + 5.

b One dense and one sparse (both categories) PZ lesion and one dense TZ lesion are GS 4 + 3 + 5.

c One dense (both categories) PZ lesion is GS 5+3+4

**Table 4:** MR Imaging: dense and sparse 2D cancerous regions separated by Gleason Score / Gleason Group and zone.

Gleason Score	GleasonGroup	Peripheral Zone		Transition Zone	
		Dense	Sparse	Dense	Sparse
≤3 + 3^a^	1	56 (23)	29 (64)	40 (37)	8 (100)
3 + 4	2	78 (32)	3 (7)	37 (34)	0 (0)
4 + 3^b^	3	44 (18)	5 (11)	25 (23)	0 (0)
3 + 5,4 + 4,5 + 3^c^	4	30 (12)	3 (7)	7 (6)	0 (0)
≥4 + 5^d^	5	38 (15)	5 (11)	0 (0)	0 (0)
**Total (N)**		**246**	**45**	**109**	**8**

Sparse 2D cancerous regions were defined as regions with cancer occupying <50 % of cross-sectional area of the region. Percentages are given in ()’s.

a Includes: GS 2 + 3: 1 dense TZ; GS 3 + 2: 1 dense PZ, 9 dense TZ, 1 sparse TZ.

b Two dense PZ and 2 dense TZ are GS 4 + 3 + 5.

c Two dense TZ are GS 3+5; 8 dense and 2 sparse PZ are GS 5+3

d Nine dense PZ are 5+4; 10 dense PZ are 5+5

**Table 5:** MR Imaging: number of dense and sparse lesions separated by Gleason Score / Gleason Group and zone.

Gleason Score	GleasonGroup	Peripheral Zone		Transition Zone	
		Dense	Sparse	Dense	Sparse
≤3 + 3	1	22 (26)	18 (60)	14 (38)	5 (100)
3 + 4	2	24 (28)	3 (10)	11 (30)	0
4 + 3	3	18 (21)	5 (17)	8 (22)	0
4 + 4, 5 + 3	4	10 (12)	2 (7)	2 (5)	0
≥4 + 5	5	11 (13)	2 (7)	2 (5)	0
**Total (N)**		**85**	**30**	**37**	**5**

Sparse lesions were defined as 3D lesions containing only sparse cancerous ROIs. Percentages are given in ()’s.

## Abbreviations:

DWI: Diffusion weighted image
DCE: MRI Dynamic contrast-enhanced MRI
GS: Gleason Score
GG: Gleason Grade Group
HGPIN: High grade prostatic intraepithelial neoplasia
mpMRI: Multiparametric MRI
PCa: Prostate Cancer
PZ: Peripheral Zone
ROI: Region of Interest
TZ: Transition Zone.
